# Willingness to Pay for Social Health Insurance in Central Vietnam

**DOI:** 10.3389/fpubh.2017.00089

**Published:** 2017-04-25

**Authors:** Lan Hoang Nguyen, Anh Thuan Duc Hoang

**Affiliations:** ^1^Institute for Community Health Research, Hue University of Medicine and Pharmacy, Hue University, Hue, Vietnam; ^2^Faculty of Public Health, Hue University of Medicine and Pharmacy, Hue University, Hue, Vietnam

**Keywords:** social health insurance, willingness to pay, contingent valuation technique, health economics, Vietnam

## Abstract

**Background:**

A social health insurance (SHI) program was implemented in Vietnam in 1992. Participation is compulsory for some groups, such as formal-sector workers and voluntary for other groups. In 2013, 68% of the total population was covered by SHI, with most enrollees from compulsory groups. Enrollment has remained low among persons whose enrollment is voluntary. As a result, households face financial risk due to high out-of-pocket payments for health care. The goal of this study is to identify willingness to pay (WTP) for the SHI scheme among persons whose enrollment is voluntary and to examine factors that influence their choice.

**Method:**

Three hundred thirty-one uninsured persons from three districts and one city of Thua Thien Hue province were interviewed face to face using a structured questionnaire. Contingent valuation technique was used to assess the WTP among the study participants. Each individual was asked to choose the maximum premium they were willing to pay for a health insurance card per year with three copayment levels of 0, 10, and 20%. Seven premium levels were offered ranging from 0 to 900,000 Vietnamese Dong (VND) (42.12 USD). The mean WTP of respondents for each scenario was estimated. Multiple linear regression analysis was used to identify factors influencing WTP for SHI.

**Results:**

The survey found that 73.1, 72.2, and 71.6%, respectively, for each copayment level, of the respondents would agree to participate in the SHI scheme and are willing to pay an annual premium of 578,926 VND (27.1 USD); 473,222 VND (22.1 USD); and 401,266 VND (18.8 USD) at the copayment levels of 0, 10, and 20%, respectively. The WTP for SHI is influenced by knowledge of SHI at all copayment levels (*p* value < 0.05). The more knowledge about SHI individuals have, the higher the WTP amount. Chronic disease was related to WTP only at a copayment level of 20% (*p* = 0.049).

**Conclusion:**

Enhanced awareness of the benefits of SHI among the population should contribute to expanding SHI coverage in Vietnam.

## Introduction

A social health insurance (SHI) scheme was introduced in Vietnam in 1992 (1). After over 20 years of operation, the SHI scheme has become an important financing source for the health-care system and contributes to improved health indicators in the country through increased access to health-care services for the beneficiaries, especially the poor and vulnerable. The insured population is divided by the government into five groups depending on contributive responsibility to the SHI fund. The contribution of the membership groups to the SHI fund is shown in Table 1 (2).

**Table 1:** **The contribution of various membership groups to the social health insurance fund [source: Midori and Hiroyuki (2)]**.

Group	Members	Premium level	Contributive responsibility
1	Workers in formal sectors	4.5% of monthly salary	Employers contribute 3%, employees contribute 1.5%
2	Pensioners People on working capacity loss allowance	4.5% of pensioner salary 4.5% of working capacity loss allowance	100% paid by the social security agency
3	The poor, minorities, and children under 6 years of age	4.5% of monthly minimum salary	100% subsidy by the government
4	The near-poor Pupils and students	4.5% of monthly minimum salary 3% of monthly minimum salary	70% subsidy by the government 30% subsidy by the government
5	Workers in informal sectors Other members of households	4.5% of monthly minimum salary 4.5% of monthly minimum salary for the first person; 70, 60, and 50% of the premium rate applicable to the first person, for the second, third, and fourth persons, respectively; 40% of this premium rate for the fifth or additional persons	100% paid by covered person

Enrollment in the SHI is referred to as “having a health insurance card.” The “card” expires each year and must be renewed in order for insurance coverage to be continued. In 2014, for group 4 and 5, the premium was set at 4.5% of the minimum salary, about 621,000 Vietnamese Dong (VND) per person per year, or about 29.07 USD (1 USD = 21,363 VND). The insurance goes into effect when the insured is provided with medical care at a health facility where he or she is registered or referred. Participants can choose to register with any public or private health facilities they wish to be responsible for their treatment, within the options given by the government. The copayment system was introduced in January 2010. The insurance covers at least 80% of the medical costs; the insured pays the rest directly to the hospital, except for groups that are subsidized 100% by the government. If insureds prefer to be treated in other health facilities, they have a higher copay of at least 40%, except in emergency cases. Any costly, technologically advanced medical treatment has a ceiling on the maximum benefit for the treatment of each episode; the ceiling is defined as the total minimum salary over 40 months. In 2012, the ceiling was equivalent to from 2,682.80 USD to 3,838.80 USD (2).

A series of incremental reforms by the government over the past several years has expanded coverage of SHI. In 1993, only 5.4% of the population was covered. By 2013, the figure had grown to about 68%. The coverage rate has been different among membership groups. The highest coverage was reported in groups that were fully subsidized by the social security agency and by the government, about 94.32 and 80.74%, respectively. In comparison, the groups that are required to make full or partial premium payments maintained a low coverage rate at 21.11%. By the end of 2012, the coverage rate for the near-poor was 25%, but only 2–5% in some regions, even though they were subsidized at 70% of the premium beginning in June 2012. Additionally, the enrollment rate of workers at private enterprises was 20–30% (3–5). It should be noted that until 2012, enrollment in the SHI schemes was by individual, not by family.

The enrollment of groups without full support is voluntary. These groups consist largely of the sector of non-poor informal workers. Families of formal-sector workers belong to these groups also since SHI for formal-sector workers is limited to individuals only. Individuals engaged in the agriculture, forestry, and fisheries sectors also belong to these groups. Under the SHI law, all of these groups are classified under the voluntary, contributory subcategory (5). Household income, the premium level for SHI, the features of the benefit package, and lack of knowledge about health insurance are considered to be reasons for low participation by these groups (5–7). There is also evidence of adverse selection among the voluntary groups. Lieberman and Wagstaff found that families of formal-sector workers have a higher probability of being enrolled in SHI if any of the family members has been ill during the past 12 months [c.f. (5)]. As a result, risk pooling is limited, and financial protection for all remains an elusive goal. Households face financial risk due to the potential for high out-of-pocket payments, which results in unequal access to health-care services (5). Achieving universal coverage is one solution to improving equality of access to health care and securing financial protection from high health-care-related costs. The Master Plan for Universal Coverage was approved by the Vietnamese Government in 2012 with the aim of expanding the coverage of SHI to 80% of the population by 2020 and reducing the out-of-pocket share of total health expenditures (5).

The study reported, here, was conducted in a central province of Vietnam with the aim of identifying willingness to pay (WTP) for the SHI scheme among persons whose enrollment is voluntary (groups 4 and 5) and examining factors affecting their WTP. The findings of the study may contribute insights about how to reach the goals set out in the government’s Master Plan.

## Materials and Methods

### Study Site

The study was conducted in Thua Thien Hue province of central Vietnam. The province had a population of 1,127,905 in 2013, of which 48.4% lives in rural areas. It is divided into nine administrative regions—one city, two towns, and six districts, consisting of 152 communes. Income per capita in 2013 was 1,700 USD/year (36,550,000 VND). The proportion of poor households was 5.3% (8). By 2014, the coverage rate for SHI in all membership groups was 81.2% of the total population of the province. The highest rates of enrollment were among the poor (100%), children under 6 years old (100%), pupils and students (93.45%), and the near-poor (87.5%) (9). For the study, four communities were selected as being representative of the major ecological regions of the province: Hue city (urban), Huong Tra town (rural), Phu Loc district (coastal and lagoon area), and Nam Dong district (mountainous area).

### Sampling and Sample Size

The sample was selected in two stages. First, 15 communes/quarters were randomly chosen from the four selected communities based on a proportional sampling method. Second, the uninsured people who met the following criteria in the chosen study areas were identified to the extent that records of the local health facilities permitted: age 18 years and over; residents of Thua Thien Hue province; classified as members of groups 4 and 5; ability to communicate; and agreement to participate in the study. The study sample did not include pupils and students because they often do not make the decision to enroll or not enroll themselves in the scheme. This list of uninsured people served as a basis for identifying potential participants. Because the government does not have detailed records of those who are not part of the SHI, the ultimate sample must be considered one of convenience. The number of people agreeing to participate in the study totaled 331.

### Data Collection

The study was cross-sectional in design. Data were collected between August and December 2014, by interviewing participants in person using a structured questionnaire. The questionnaire was in three parts: part 1 was a demographic and socioeconomic profile of the respondent, part 2 asked questions about the individual’s knowledge of SHI, and part 3 was about the levels of WTP for the SHI annual premium.

Knowledge about SHI was measured by 13 questions about principles of SHI and the duties of and benefits to insureds under the scheme. The maximum possible score for knowledge was 42. Knowledge was rated as “good” for scores of 22 and above; scores of 21 and below were characterized as “not good.” The questionnaire was pre-tested on a group of 10 people in Hue city and revised before implementing data collection.

### Eliciting WTP

Contingent valuation was used to elicit the WTP of participants using the payment card technique (10). A health insurance scheme with ongoing benefits and responsibilities was explained, and three scenarios with different copayment levels of 0, 10, and 20% were introduced to participants. For each scenario, seven premium levels were listed including 0 VND; 150,000 VND (7.02 USD); 300,000 VND (14.04 USD); 450,000 VND (21.06 USD); 600,000 VND (28.08 USD); 750,000 VND (35.10 USD); and 900,000 VND (42.12 USD). Premium levels were presented in both VND and USD. The exchange rate used was that in effect on July 15, 2014 (1 USD = 21,363 VND). Respondents were asked to check all monetary values they would be willing to pay annually for SHI, up to and including the maximum amount. Respondents who chose only the value 0 VND were asked a follow-up question to confirm that their answer reflected their WTP rather than a preference not to join the SHI scheme at any premium level.

### Data Analysis

Of the seven possible premium options for each copayment, the maximum WTP was used to calculate the mean and median WTP of respondents for copayments at each of the 0, 10, and 20% levels. The study expected to test a hypothesis that there is positive relationship between people’s WTP for SHI on the one hand, and the socioeconomic characteristics of participants and their knowledge about SHI at different copayment levels on the other. A linear multiple regression model was used to test the hypothesis. In the model, WTP for SHI was considered a dependent variable. Predictor variables included socioeconomic characteristics (such as age, sex, place of residence, marital status, years of schooling, occupation, family size, and household income), the participant’s having a chronic disease, knowledge of SHI, and the perceived quality of the health facility that the insureds registered. These were factors having influence on WTP for SHI as shown in previous studies (11–15). All categorical predictor variables were converted to a dichotomous format with two levels (0 and 1). For variables with multiple levels, such as place of residence and occupation, the process of creating dichotomous variables was based on the principle that a categorical variable with *k* levels would be transformed into *k* − 1 variables, each with two levels. For example, occupation had five dichotomous variables. An alpha level of 0.05 was considered statistically significant.

## Results

### Characteristics of Respondents

Tables 2 and 3 describe the demographic and socioeconomic characteristics of the respondents. They show that all the respondents were of Kinh ethnicity and half of them were male. Participants aged 25–50 years were the majority, representing 60.5% of the sample. Very few of the respondents lived in mountainous areas, while 68.3% were residents of rural or coastal areas. Primary or secondary school was the most frequent educational attainment levels of respondents. All of the respondents worked in the informal sector. About 2% of the interviewees’ households were classified as near-poor. The average household size was five. More than half of respondents had enrolled in the SHI scheme sometime in the past.

**Table 2:** **Demographic characteristics of respondents**.

Characteristics		*N*	Percent
Sex	Males Females	168 163	50.8 49.2
Age (years)	<25 25–50 >50	21 200 110	6.3 60.5 33.2
Average age		44 ± 12 (Min: 18; Max: 79)	
Ethnic group	Kinh Minority	331 0	100.0 0.0
Place of residence	Mountain Rural or coastal area Urban	13 226 92	3.9 68.3 27.8
Total		331	100

**Table 3:** **Socioeconomic characteristics of respondents**.

Characteristics		*N* (331)	Percent
Education	Illiterate Primary school Secondary school High school Higher	30 99 139 48 15	9.1 29.9 42.0 14.5 4.5
Occupation	Farmer Small trader Craftsman Self-employed Housewife Other	114 58 18 77 11 53	34.4 17.5 5.4 23.3 3.3 16.0
Marital status	Married Divorced/widowed/single	280 51	84.6 15.4
Economic condition	Near-poor Normal	8 323	2.4 97.6
Monthly average income per capita (SD)		1,214,684 Vietnamese Dong (VND) (836,546) 56.9 USD (39.20)	
Number of persons/household		5 ± 2 (Min: 1; Max: 10)	
Participated in social health insurance in the past	Yes No	176 155	53.2 46.8
Has chronic disease	Yes No	63 268	19 81

Reasons for refusal to join the SHI scheme are presented in Table 4. The two main reasons were lack of money to buy a health insurance card and a perception that health insurance was not necessary because the respondent was healthy.

**Table 4:** **Reasons for unwillingness to join the social health insurance (SHI) scheme**.

Reason	*N* (331)	Percent
Not enough money to buy health insurance card	232	70.1
No illness/healthy	104	31.4
Complicated SHI administration	12	3.6
Poor health-care quality	2	0.6
Other	13	3.9

Almost all respondents had heard of the SHI scheme. However, 87.9% of participants lacked detailed knowledge about how SHI works and what its benefits and responsibilities are (Table 5). The average score of respondents’ knowledge about SHI was 12.4, much lower than the maximum possible score of 42. Information related to primary care facilities for insureds, collection agents for SHI, the categories of insureds, and members in voluntary groups were the features known by the largest percentage of respondents. However, only a few respondents clearly knew the principles of SHI as well as they did the financial incentive policy for family enrollment.

**Table 5:** **Knowledge of social health insurance (SHI) of respondents**.

	*N* (331)	Percent
**Heard of SHI information**		
Yes	304	91.8
No	27	8.2
**Overall knowledge of SHI**		
Good	40	12.1
Not good	291	87.9
**Right answers about SHI**		
Principles of SHI	27	8.2
Categories of insureds	210	63.4
Which people are in compulsory groups	64	19.3
Which people are in voluntary groups	175	52.9
Premium levels	113	34.1
Premium rates for members of household	35	10.6
Copayment levels	58	17.5
Benefit package	145	43.8
Services uncovered by SHI	67	20.2
Primary care facilities	307	92.7
Formalities to use SHI	136	41.1
Duties of insured	119	36.0
Collection agents for SHI	288	87.0

### WTP for SHI

**Figure 1:**
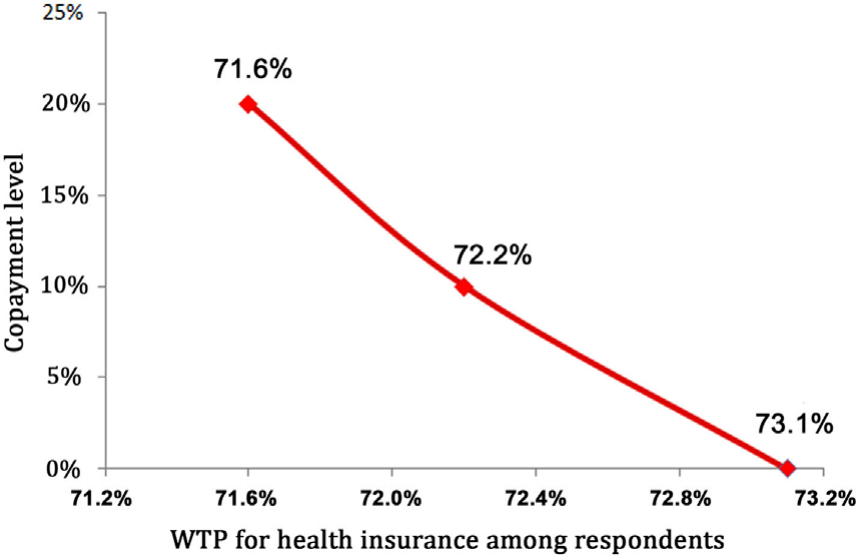
**Willingness to pay (WTP) for social health insurance with different copayment levels**.

The number of respondents willing to pay for the SHI scheme decreased with an increase in the copayment level. More than 73 (73.1), 72.2, and 71.6% of respondents expressed WTP for SHI at copayment levels of 0, 10, and 20%, respectively (Figure 1).

Willingness to pay for the annual health insurance card at different copayment levels is presented in Table 6. The mean WTP was lower at higher copayment levels.

**Table 6:** **Willingness to pay (WTP) for annual health insurance card at different copayment levels**.

WTP	Copayment		
	0%	10%	20%
Mean (SD)	578,926 VND (187,166)	473,222 VND (172,642)	401,266 VND (161,029)
	27.10 USD (8.76)	22.15 USD (8.08)	18.78 USD (7.54)
Median	600,000 VND	450,000 VND	450,000 VND
	28.08 USD	21.06 USD	21.06 USD
Minimum	150,000 VND	150,000 VND	150,000 VND
	7.02 USD	7.02 USD	7.02 USD
Maximum	900,000 VND	900,000 VND	900,000 VND
	42.12 USD	42.12 USD	42.12 USD

### Factors Influencing WTP

Table 7 shows the results of the multiple linear regression analysis. The strength of the relationship between the models and the response variables is low with *R*-squared of 0.13, 0.21, and 0.17 for the models with copayment levels of 0, 10, and 20%, respectively. Knowledge about SHI influenced the decision-making process for WTP for SHI at all copayment levels. As the knowledge score increased, WTP levels increased by 5,199 VND (0.24 USD), 7,041 VND (0.33 USD), and 4,719 VND (0.22 USD) at the copayment levels of 0, 10, and 20%, respectively. Chronic disease was related to WTP only for a copayment of 20% (*p* = 0.049). Respondents with chronic disease were willing to pay for SHI 64,531 VND (3.02 USD) less than those who were healthy. The study did not find a significant association between WTP and any other variables, including age, sex, household size, income per capita, occupation, or quality of health-care services under any of the SHI scenarios proposed.

**Table 7:** **Factors influencing willingness to pay for social health insurance (SHI)**.

Predictors	Copayment 0%			Copayment 10%			Copayment 20%		
	*B*	SE	*p-*Value	*B*	SE	*p-*Value	*B*	SE	*p-*Value
Age (years)	111.8	1,337.5	0.933	−6.9	1,185.7	0.995	−31.2	1,137.5	0.978
Sex^a^ (male = 1)	−3,493.1	32,452.3	0.914	14,160.9	28,769.6	0.623	−3,013.6	27,600.7	0.913
Married status^a^ (spouse = 1)	24,672.3	43,997.2	0.576	19,102.5	39,004.3	0.625	39.6	37,419.6	0.999
Years of schooling	7,623.5	4,494.6	0.092	5,820.2	3,984.6	0.146	5,981.2	3,822.7	0.120
Place of residence^b^ [Mountain (ref.)]									
Rural and coastal area	−21,636.2	75,157.0	0.774	−52,864.7	66,628.1	0.429	−44,712.5	63,920.9	0.485
Urban	−2,686.5	78,682.2	0.973	−3,301.5	69,753.2	0.962	−7,118.92	66,919.1	0.915
Occupation^b^ [Housewife (ref.)]									
Farmer	54,698.8	82,368.5	0.508	49,788.9	73,021.2	0.496	37,125.8	70,054.3	0.597
Small trader	61,917.0	85,112.7	0.468	49,116.9	75,454.0	0.516	42,396.5	72,388.3	0.559
Craftsman	26,280.7	98,552.9	0.790	42,385.5	87,368.9	0.628	43,351.3	83,819.1	0.606
Self-employed	101,062.7	86,547.9	0.245	93,499.1	76,726.3	0.225	67,582.6	73,608.9	0.360
Other	90,320.1	87,459.7	0.303	85,773.4	77,534.7	0.270	85,397.5	74,384.4	0.253
Number of household members	6,229.6	10,246.1	0.544	3,329.9	9,083.4	0.714	536.1	8,714.3	0.951
Average income per capita	0.002	0.003	0.546	0.000	0.003	0.894	0.002	0.003	0.514
Chronic disease^a^ (yes = 1)	−55,761.9	38,218.8	0.146	−61,295.2	33,881.7	0.072	−64,530.5	32,505.1	0.049
Total score on knowledge of SHI	5,198.6	2,175.1	0.018	7,041.3	1,928.3	<0.001	4,719.1	1,849.9	0.012
Total score of quality of health facility	−2,730.6	5,758.4	0.636	−1,084.6	5,104.9	0.83	−2,859.1	4,897.5	0.560
Constant	390,117.9	153,552.2	0.012	301,909.6	136,126.8	0.03	323,932.0	130,595.9	0.014

*^a^Dummy variables with two levels (0 and 1)*.

*^b^Dummy variables with multiple levels*.

## Discussion

### Main Characteristics of Study Subjects

The respondents were uninsured persons at the time of the study. All of them worked in the informal sector, and they were classified as being part of the voluntary health insurance groups. Surprisingly, eight respondents who belonged to the near-poor group accounted for 2.4% of the study sample, although their premium is covered by the local and national governments, and their cost is only 5–10% of the current premium. The vast majority of respondents did not report having any chronic disease. Average income per capita was reported to be lower than that of the province in general (1.2 million VND/month versus 3.0 million VND/month) (8).

Lack of financial resources and present good health were the main reasons why the respondents did not enroll in the SHI (Table 3). The findings were similar to those of previous research in Vietnam (7, 11, 16). These findings show that the purposes and principles of SHI are not yet well understood by this portion of the population. Respondents’ knowledge of SHI was poor, although almost all of them had heard about the scheme (Table 5). This is consistent with the report of Aparnaa, et al. The authors found that many Vietnamese people did not have a good understanding of the benefits of the insurance, nor of the limits of coverage and copayment policies. Thus, they neither did enroll in SHI nor use the health insurance card even when enrolled (5).

This result indicates an existing communication challenge. Either the communication methods used did not attract the interest of the population or its contents were not appropriate. Communication strategy, modality, and contents should be tailored for each respondent group in order to improve awareness, with the goal of increasing enrollment in SHI.

### WTP for Enrollment in the SHI System

The number of respondents willing to pay for enrollment in SHI decreased with an increase in copayment levels. Similarly, the WTP for annual SHI card decreased as the copayment levels increased (Table 6). This accords with the findings of Bärnighausen, who studied uninsured informal- sector workers in Wusan, China (12). This author reported that average WTP increased significantly when any one of the copayments for the basic health insurance was removed. The findings of the study reported here revealed that the mean WTP for a health insurance card per year was 578,926 VND (27.10 USD), 473,222 VND (22.15 USD), and 402,266 VND (18.78 USD), for the copayment levels of 0, 10, and 20%, respectively. All of these results are lower than the current premium for SHI (621,000 VND or 29.07 USD). Respondents reported being healthy; hence, the value of SHI was not perceived to be significant.

A study on the enrollment in SHI of farmers of Vu Ngoc Huyen in Thai Binh, a province in northern Vietnam, found that over 80% of farmers agreed to join the SHI scheme with an average WTP of 573,000 VND at a copayment of 20% (11). Another study in Ha Tinh, a province in central Vietnam, reported that people needed a decrease in the voluntary health insurance premium before they would enroll (7). Although the topics of investigation of previous studies in Vietnam were different from those of the study reported here, the findings of these previous studies imply that people will not enroll in the SHI if the premium is not reduced to affordable levels.

From the policy viewpoint, the WTP data could be used to estimate an acceptable premium for SHI. The low WTP elicited could affect SHI’s objective of financial risk protection because of the resulting limits on risk pooling. This study was conducted after enrollment criteria were changed in 2012. The new policy stipulated that premium levels would be reduced with an increase in the number of enrollees in a household (Table 1) (2). This encourages family-based enrollment. The regulation was intended to respond to both the need to expand the coverage of SHI and strengthen risk pooling. However, family enrollment implies greater absolute expenditures by households on SHI, even if the per person cost decreases.

The WTP of 578,926 VND (27.10 USD) per year for SHI without a copayment suggests that annual expenditures for health were expected to be only 4% of the total income of the average household. This is higher than reported by a study in Ghana which revealed that participants were willing to pay 1.9 to 2.5% of their annual incomes for health insurance premiums (13). The results of a study by Tesfamichael, et al. in Ethiopia showed results similar to those of the study here. Also in Ethiopia, Tesfamichael found that 47.1% of teachers who were asked were willing to pay 4% of their monthly salaries to join the SHI system (14). Authors have offered differences in the various schemes’ benefit packages and in participants’ past experience of high health-care expenditures as reasons for varying WTPs (13). In our study, the benefit package introduced to participants was the same as the current health insurance card coverage in Vietnam. It is understood that the respondents’ WTP values represent the amount they are willing to invest to avoid the potentially catastrophic costs of health care.

### Factors Related to WTP for the Health Insurance Scheme

Multiple linear regression analysis showed that knowledge about SHI is significantly and positively related to the WTP value of respondents in the three copayment scenarios. Chronic disease shows a negative relationship of WTP for SHI in the scenario of a 20% copayment (*p* = 0.049) (Table 7).

A high level of awareness about SHI is a prerequisite for a successful SHI scheme. This is consistent with results obtained by previous studies around the world, as well as in Vietnam. In Ghana, when health insurance policy was first introduced in 1997, many people were willing to join the scheme and expected a successful system (13). In Ethiopia, Tesfamichael found that teachers who had heard about the health insurance scheme were 2.5 times more willing to pay for it than those who had never heard of it (14). Vu Ngoc Huyen reported similar findings for Vietnam in Thai Binh. According to the author, farmers who understood SHI had higher WTP values than their counterparts who did not know anything about the policy (11). In the current study, knowledge of respondents about SHI is the only predictor of WTP which presents at all copayment levels. Those with a good understanding of the principles of financial protection offered by SHI realized that enrollment in the scheme is a worthwhile investment. These results confirm again the importance of a targeted communication program for the expansion of SHI. Thus, sensitization about SHI entitlements should be achieved in all population groups through the use of relevant marketing strategies.

The study found a negative relationship between chronic disease and WTP for SHI in the scenario of a 20% copayment. Respondents with chronic disease tended to have lower WTP values than those without chronic disease (Table 7). On the contrary, Mathiyazhagan revealed that households experiencing illness were willing to pay 2.72 times more for a proposed rural health insurance scheme than those without illness (15). Another study in India reported that frequency of hospitalization tended to increase WTP for a health insurance scheme (17). The authors believed that those with chronic diseases often face high risks of large health-care costs, and therefore, they are interested in a health insurance scheme to avoid these costs. Indeed, the positive association between incurring medical costs in the past and WTP has been confirmed in many previous studies (12, 15, 17, 18). In studies among people in voluntary groups in Vietnam, individuals who were ill during the previous 12 months or the previous 4 weeks had an increased motivation to enroll in SHI. The authors concluded that this was evidence of adverse selection (5). In our study, the health problems might be not as serious as those in the previous studies, or the patients may have had the option of employing informal self-treatment methods, such as utilizing herbs, as replacement solutions for using health services provided at health facilities. This suggests the possibility that participants with chronic disease in our study had not faced a previous experience of high health-care expenses, lowering their WTP values. The result in this study also indicates that the problem of adverse selection may not be a serious concern in the studied areas.

Surprisingly, although lack of financial resources is also a principal reason for refusal to join SHI reported by participants in this study, income of household was not related to WTP for any of the copayment levels proposed in the study. This finding might be affected by the subsidy for the near-poor group (Table 1). This could imply that both the poor and the more well-off expected to join SHI. Again, there would be no serious adverse selection problem.

### Limitations

This study has several limitations. First, the sample was small and created out of convenience, given the lack of a complete list of those who are not enrolled in the SHI scheme. Bias selection could be potential problem. The group representing students was excluded from the study sample, so issues related to their lack of enrollment were not investigated. However, it should be noted that students are usually not the people in the household who decide to join SHI or pay for the family’s enrollment. Second, the highest level offered for the payment card perhaps did not reflect the highest premium that some respondents would be willing to pay. Finally, the study was conducted in the Thua Thien Hue province, an area that has achieved a high proportion of enrollment in the SHI scheme. This could limit the ability to generalize the study findings. However, it should be noted that the WTP values found in the study are similar to those of other studies of communities with similar incomes per capita (11). It can be inferred that these WTP values are applicable to communities with similar socioeconomic characteristics.

## Conclusion

Uninsured people in Vietnam are willing to pay less in premiums for SHI than the amount of the current premiums in the existing SHI scheme. Knowledge of the benefits of SHI is a common factor influencing WTP for the scheme. The results suggest that educating the population about the benefits of SHI is essential for increasing enrollment. Information and communication strategies should be diversified and targeted to appeal to different population groups.

## Ethics Statement

Approval for the study proposal was obtained from Hue University of Medicine and Pharmacy. In addition, approval for implementing the study at the sites was obtained from Health Services in Thua Thien Hue province. The interview of study subjects was performed with their permission after they were given adequate information about the study in a consent form. Ethical principles of research on human subjects such as autonomy, beneficence, and justice were introduced in this form. The vulnerable populations did not present in our study.

## Author Contributions

LN developed the idea of the study, proposal, and tool for data collection, analyzed the data and wrote the report. AH organized data collection in study field, entered the data.

## Conflict of Interest Statement

The authors declare that the research was conducted in the absence of any commercial or financial relationships that could be construed as a potential conflict of interest.
